# Downregulation of PSAT1 inhibits cell proliferation and migration in uterine corpus endometrial carcinoma

**DOI:** 10.1038/s41598-023-31325-0

**Published:** 2023-03-11

**Authors:** Min Wang, Song Yue, Zhu Yang

**Affiliations:** Department of Gynecology and Obstetrics, The Second Affiliated Hospital of Chongqing Medical University, Chongqing, 400010 China

**Keywords:** Cancer genetics, Oncogenes, Tumour biomarkers, Computational biology and bioinformatics, Biomarkers, Diseases, Oncology

## Abstract

Phosphoserine aminotransferase 1 (PSAT1) has been associated with the occurrence and development of various carcinomas; however, its function in uterine corpus endometrial carcinoma (UCEC) is unknown. We aimed to explore the relationship between PSAT1 and UCEC using The Cancer Genome Atlas database and functional experiments. PSAT1 expression levels in UCEC were employed using the paired sample t-test, Wilcoxon rank-sum test, the Clinical Proteomic Tumor Analysis Consortium database, and the Human Protein Atlas database, while survival curves were constructed using the Kaplan–Meier plotter. We performed Gene Ontology (GO) and Kyoto Encyclopedia of Genes and Genomes (KEGG) enrichment analysis to explore the possible functions and related pathways of PSAT1. Furthermore, single-sample gene set enrichment analysis was performed to detect the relationship between PSAT1 and tumor immune infiltration. StarBase and quantitative PCR were used to predict and verify the interactions between miRNAs and PSAT1. The Cell Counting Kit-8, EdU assay, clone formation assay, western blotting and flow cytometry were used to evaluate cell proliferation. Finally, Transwell and Wound healing assays were used to assess cell invasion and migration. Our study found that PSAT1 was significantly overexpressed in UCEC, and this high expression was associated with a worse prognosis. A high level of PSAT1 expression was associated with a late clinical stage and, histological type. In addition, the results of GO and KEGG enrichment analysis showed that PSAT1 was mainly involved in the regulation of cell growth, immune system and cell cycle in UCEC. In addition, PSAT1 expression was positively correlated with Th2 cells and negatively correlated with Th17 cells. Furthermore, we also found that miR-195-5P negatively regulated the expression of PSAT1 in UCEC. Finally, the knockdown of PSAT1 resulted in the inhibition of cell proliferation, migration, and invasion in vitro. Overall, PSAT1 was identified as a potential target for the diagnosis and immunotherapy of UCEC.

## Introduction

Uterine corpus endometrial carcinoma (UCEC) is the most common cancer of the female reproductive organs^1^ in the developed world^2–4^, and its incidence and mortality rate are increasing annually, while the age of onset is decreasing. Many UCEC patients can recover with active treatment^5^; however, a small number of patients miss the optimum treatment window due to late diagnosis^6^, which eventually leads to poor prognosis^7^. An effective diagnostic marker could help with early disease detection and, thus timely treatment measures, thereby improving therapeutic efficacy and survival. Therefore, it is crucial to identify differential molecular markers of UCEC to increase the survival rate of UCEC^8–13^ patients.

Phosphoserine aminotransferase 1 (PSAT1) is a pivotal enzyme that governs the production of two metabolites^14^, serine and α-ketoglutarate, which are involved in carbon metabolism and the tricarboxylic acid cycle, respectively^15^. In many tumors,^14,16–19^ PSAT1 plays a key role in its progression. PSAT1 enhances cell proliferation in estrogen receptor-negative breast cancer^20,21^, while its overexpression promotes the metastasis of lung adenocarcinoma^22^. Moreover, PSAT1 is a promising prognostic marker for lower-grade gliomas^23^. PSAT1 is also associated with the growth and prognosis of epithelial ovarian cancer^18^; however, it’s still unclear how PSAT1 contributes to UCEC^24^.

According to our study, we speculated that PSAT1 could have an important function in UCEC and aimed to explore its efficacy as a marker in the diagnosis and prognosis of UCEC.

## Results

### Expression profiles of PSAT1 in pan cancer analysis and UCEC

**Figure 1:**
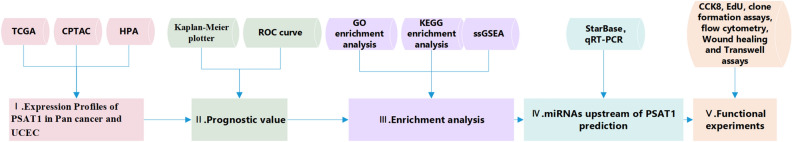
Study flow-chart.

**Figure 2:**
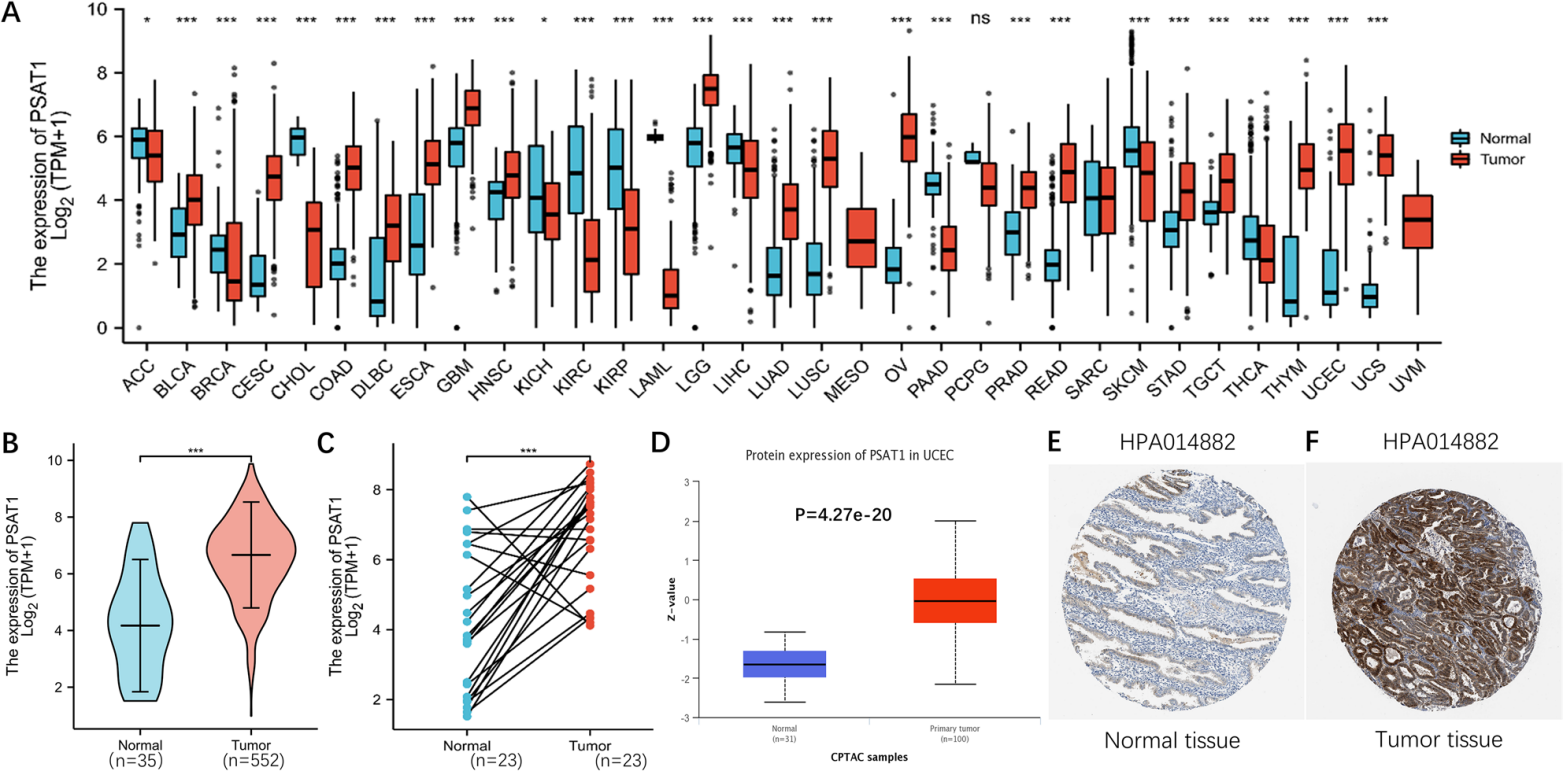
Expression Profiles of PSAT1 in Pan cancer and UCEC. (**A**) PSAT1 expression levels in Pan cancer and matched normal tissues. (**B**) PSAT1 expression levels in UCEC and normal endometrial tissues. (**C**) PSAT1 expression levels in UCEC and paired normal endometrial tissues. (**D**) The protein expression levels of PSAT1 in UCEC and normal endometrial tissues based on CPTAC database. Immunohistochemical results of PSAT1 in normal endometrial tissues (**E**) and UCEC tissues (**F**) from the HPA database. *p < 0.05, ***p < 0.001, ns, P > 0.05.

The study process is shown in Fig. 1. The Cancer Genome Atlas (TCGA) was used to determine the mRNA expression levels of PSAT1 in different cancers. Among the 33 cancer types we evaluated, PSAT1 was significantly highly expressed in 18 cancers, especially UCEC (Fig. 2A). A significant increase in PSAT1 expression was observed in 552 UCEC tissues compared to 35 normal endometrial tissues (Fig. 2B). And the expression levels of PSAT1 were also higher in the 23 tumor tissues compared to the paired normal tissues (Fig. 2C). PSAT1 protein expression was much higher in UCEC than in normal tissues, as demonstrated by the Clinical Proteomic Tumor Analysis Consortium (CPTAC) database (Fig. 2D). Furthermore, results from the Human Protein Atlas (HPA) database further confirmed that PSAT1 was significantly overexpressed in UCEC at the protein expression level. (Fig. 2E,F).

### The prognostic and diagnostic value of PSAT1 expression in UCEC

**Figure 3:**
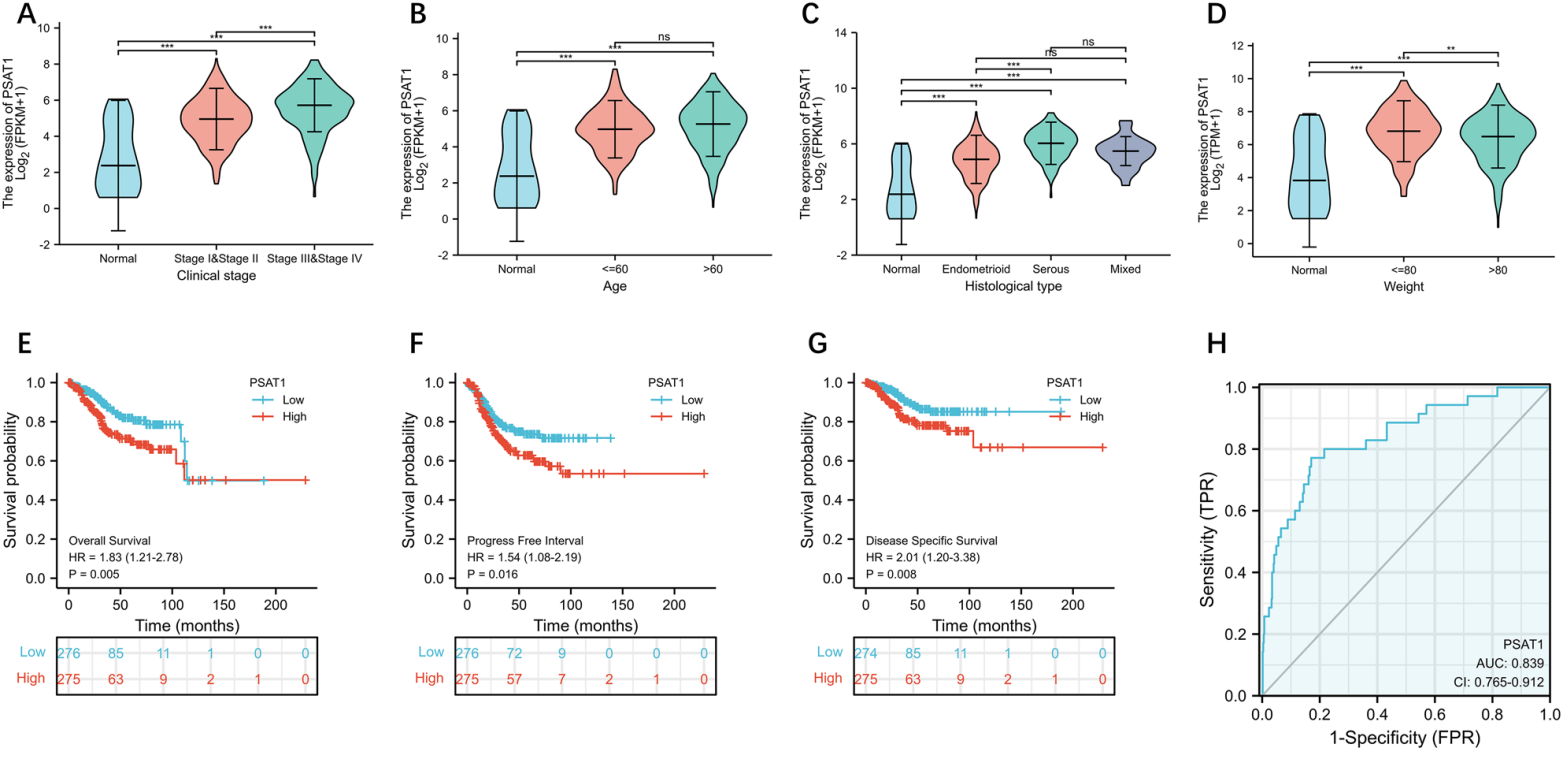
The prognostic and diagnostic value of PSAT1 expression in UCEC. Association of PSAT1 expression level with clinical stage (**A**), age (**B**), histological type (**C**) and weight (**D**) in UCEC. Survival curves of OS (**E**), PFI (**F**) and DSS (**G**). (**H**) A diagnostic ROC curve analysis evaluating the performance of PSAT1 for UCEC diagnosis. *p < 0.05, **p < 0.01, ***p < 0.001, ns, P > 0.05.

The Kruskal–Wallis rank-sum test revealed a strong correlation between the expression level of PSAT1 and the clinical stage, age, histological type and weight (Fig. 3A–D). Furthermore, high-PSAT1 expression was significantly correlated with overall survival (Fig. 3E), progression free interval (Fig. 3F), and disease specific survival (Fig. 3G) in UCEC. In addition, the diagnostic ROC curve showed that the area under the curve of PSAT1 was 0.839 (Fig. 3H), indicating that PSAT1 may be a promising tumor diagnostic marker for UCEC.

### Identification of differentially expressed genes (DEGs) between high- and low- PSAT1 expression groups, functional enrichment analysis, and predicted signaling pathways

**Figure 4:**
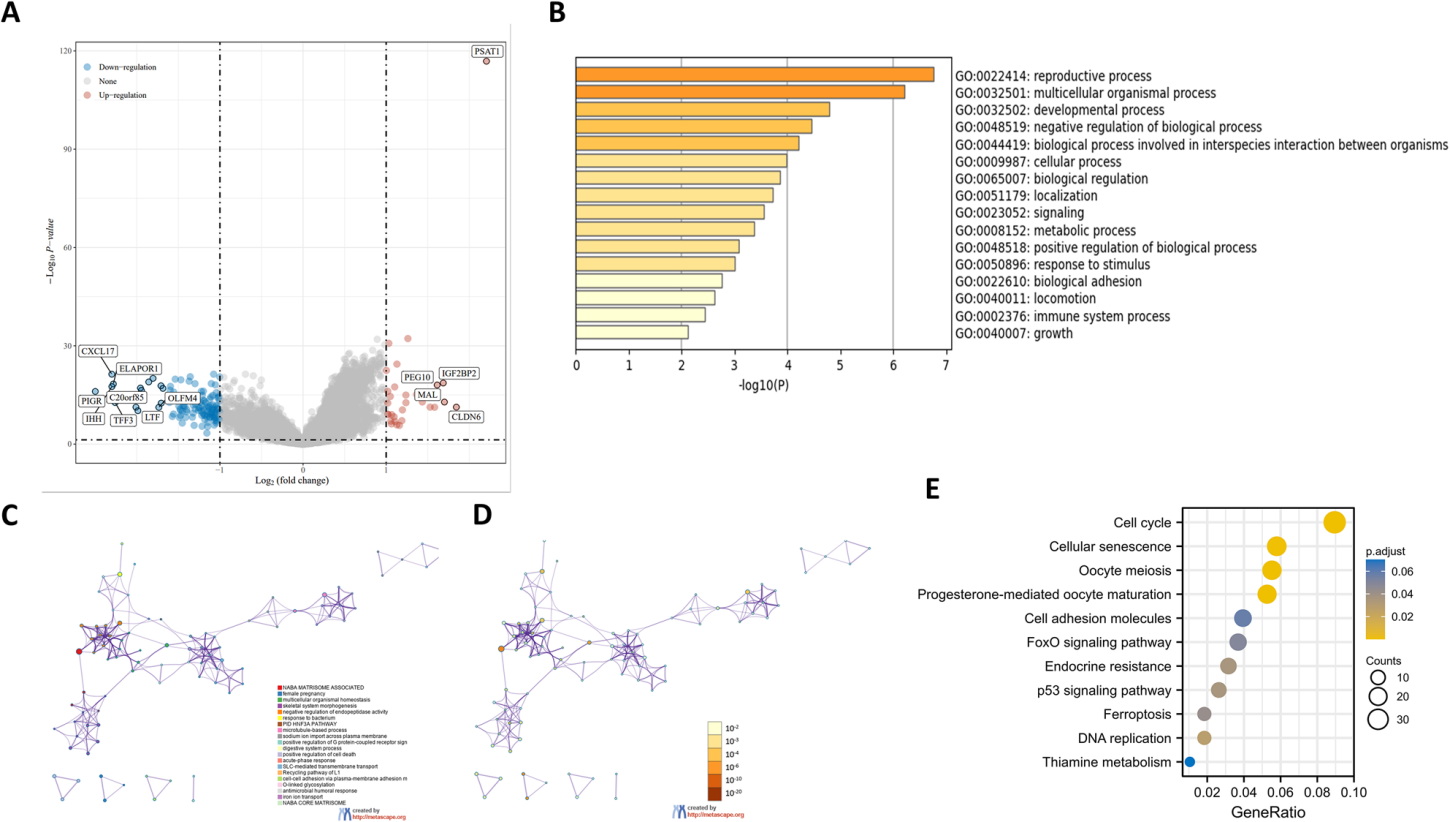
DEGs and enrichment analysis. (**A**) Volcano Plot of differentially expressed genes (DEGs). (**B**–**D**) GO enrichment analysis performed by the Metascape database 2.0. (**E**) KEGG enrichment analysis to identify the key pathways related to PSAT1 in UCEC.

A total of 148 DEGs were identified between the high- and low- PSAT1 expression groups, including 32 upregulated and 116 downregulated genes in the high-expression group (Fig. 4A). To better understand the functional implications of PSAT1 in UCEC among the 148 DEGs, Gene Ontology (GO) enrichment analysis^25,26^ was performed. The most relevant GO enrichment functions were metabolic, immune system, reproductive, and growth processes. (Fig. 4B–D). In addition, we identified critical pathways associated with PSAT1 in UCEC using Kyoto Encyclopedia of Genes and Genomes (KEGG) enrichment analysis^27–29^. The results revealed that the cell cycle and cellular senescence were significantly enriched (Fig. 4E). Some studies reported the important role of PSAT1 in the regulation of cell cycle^30^, and the results of KEGG enrichment analysis showed the highest correlation between the cell cycle pathway and the expression of PSAT1 in UCEC. Therefore, we speculated that PSAT1 might be involved in the regulation of cell cycle in UCEC.

### Correlation between PSAT1 expression and immune infiltration

**Figure 5:**
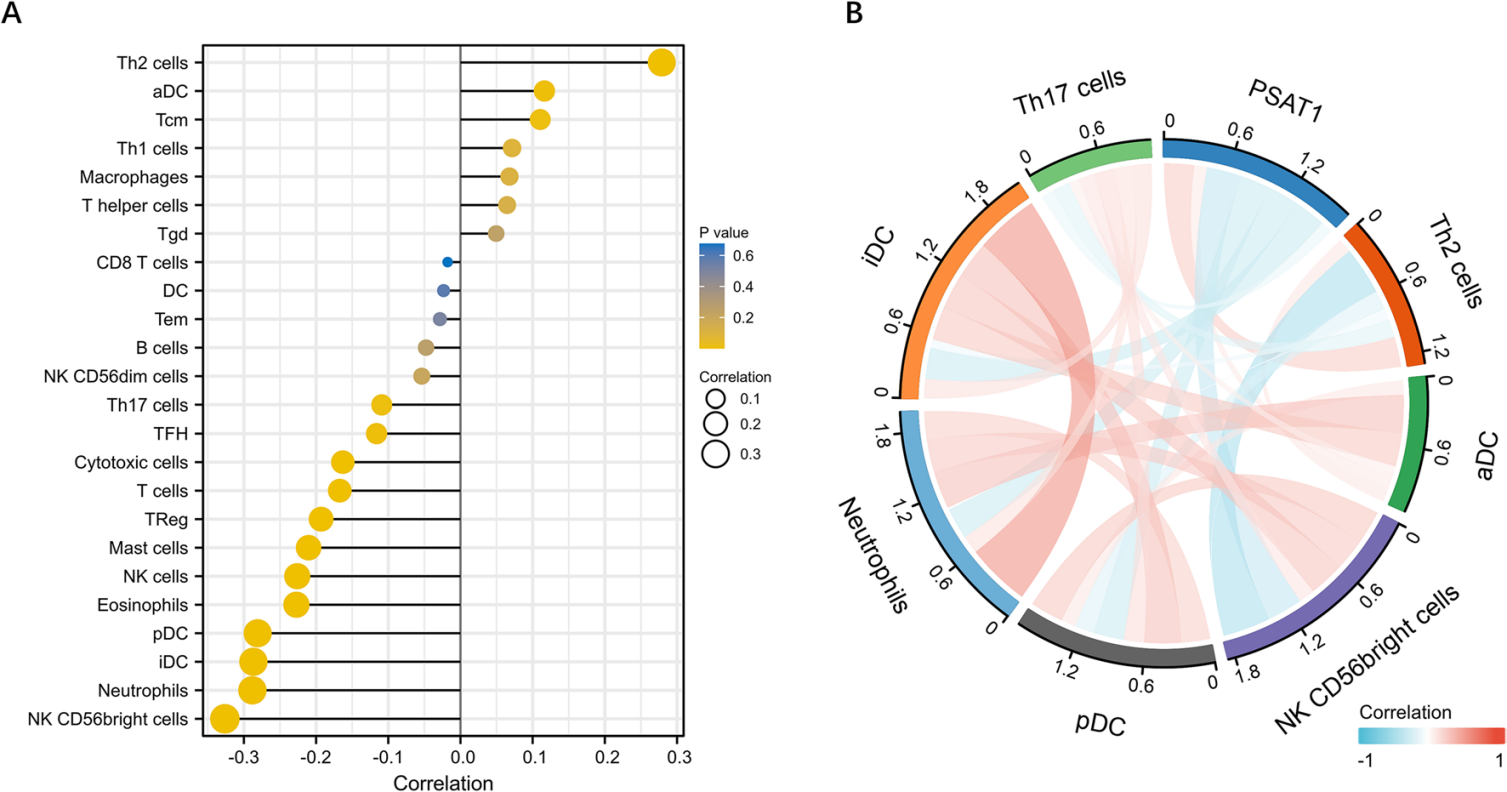
ssGSEA of PSAT1 in UCEC. (**A**) Correlation between the relative abundances of 24 immune cells and PSAT1. The size of dots represents the absolute value of Spearman R. (**B**) The immune cells with clear correlation were visualized in the form of a chord diagram.

Considering that PSAT1 may be involved in the immune system response according to GO enrichment analysis, a single-sample gene set enrichment analysis (ssGSEA) was used to examine the relationship between PSAT1 mRNA expression levels and immune cell infiltration. As shown in Fig. 5A,B, PSAT1 mRNA expression correlates with immune cell infiltration. The results revealed that the PSAT1 mRNA expression levels were positively related to the infiltration of type-2 T helper (Th2) cells and negatively related to the infiltration of type-17 T helper (Th17) cells.

### Prediction and verification of miRNAs upstream of PSAT1

**Figure 6:**
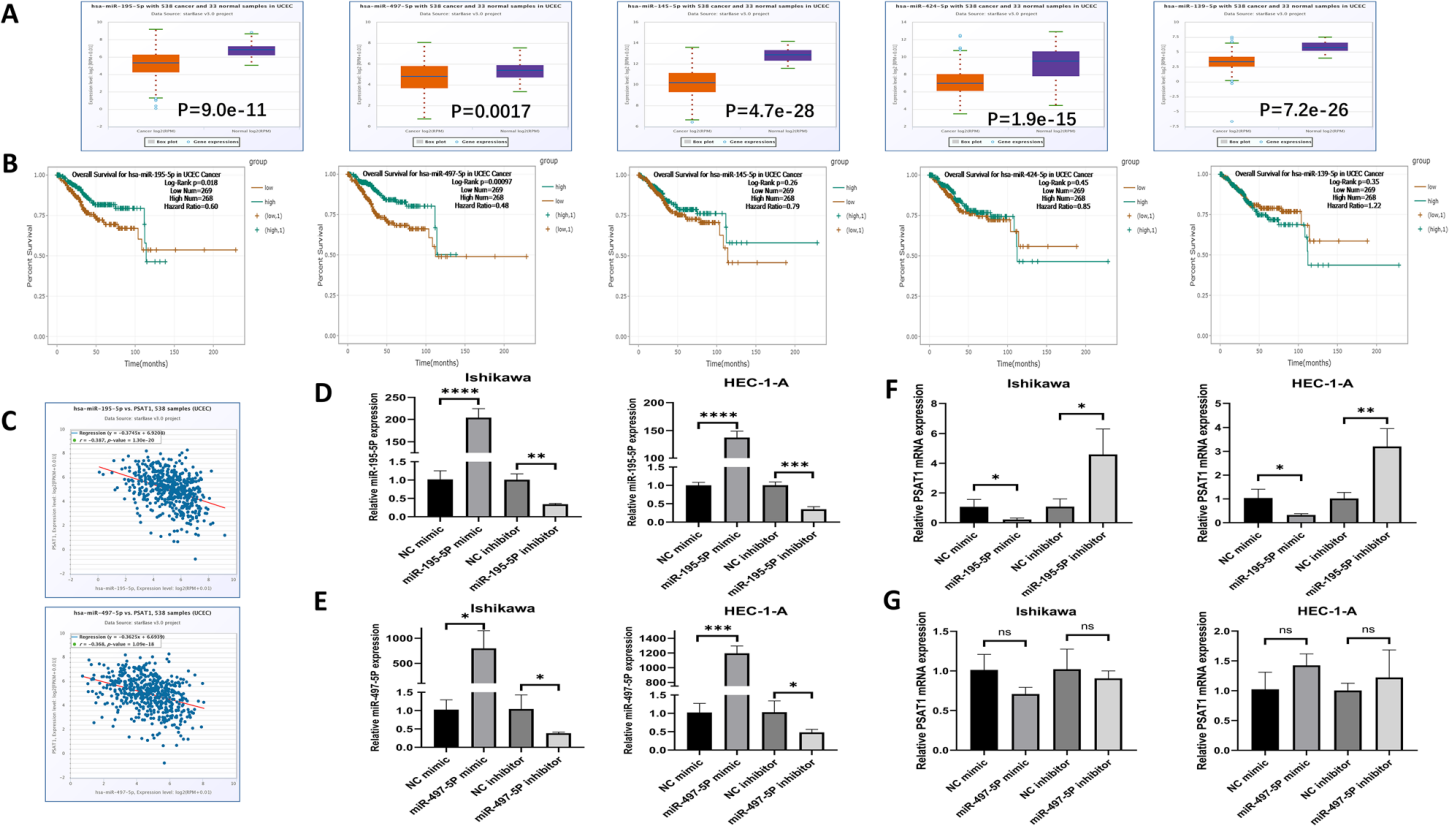
Prediction and analysis of miRNAs upstream of PSAT1. (**A**) The expression level of has-miR-195-5P, hsa-miR-497-5P, hsa-miR-145-5P, hsa-miR-424-5P and hsa-miR-139-5P in UCEC. (**B**) Survival analysis of hsa-miR-195-5P, hsa-miR-497-5P, hsa-miR-145-5P, hsa-miR-424-5P and hsa-miR-139-5P in UCEC. (**C**) Correlation analysis between hsa-miR-195-5P, hsa-miR-497-5P and PSAT1. (**D**) The transfection efficiency of miR-195-5P mimic/inhibitor in Ishikawa and HEC-1-A cells. (**E**) The transfection efficiency of miR-497-5P mimic/inhibitor in Ishikawa and HEC-1-A cells. (**F**) The relative mRNA expression level of PSAT1 after Ishikawa and HEC-1-A cells were transfected with miR-195-5P mimic/inhibitor. (**G**) The relative mRNA expression level of PSAT1 after Ishikawa and HEC-1-A cells were transfected with miR-497-5P mimic/inhibitor. *p < 0.05, **P < 0.01, ***p < 0.001, ****P < 0.0001, ns, P > 0.05.

We used StarBase to predict possible miRNAs upstream of PSAT1 in UCEC. Five possible miRNAs were predicted, including hsa-miR-195-5P, hsa-miR-497-5p, hsa-miR-145-5P, hsa-miR-424-5P and hsa-miR-139-5P. The results showed that the expression levels of these five miRNAs were lower in UCEC compared to those in normal endometrial tissues (Fig. 6A). Moreover, we analyzed the association of these five miRNAs with UCEC prognosis (Fig. 6B); however, only two miRNAs (hsa-miR-195-5P and hsa-miR-497-5p) were strongly negatively correlated with the prognosis of patients with UCEC. We analyzed the correlation between these miRNAs and PSAT1 in UCEC and demonstrated that hsa-miR-195-5P and hsa-miR-497-5p levels were negatively correlated with PSAT1 expression (Fig. 6C). Subsequent transfection experiment of a miR mimic/inhibitor showed that PSAT1 was negatively regulated by miR-195-5P, whereas miR-497-5P had no significant regulatory effect on Ishikawa or HEC-1-A cells—human endometrial cancer cell lines (Fig. 6D–G). Therefore, we speculated that miR-195-5P is an upstream regulatory gene of PSAT1 in UCEC.

### PSAT1 promotes Ishikawa and HEC-1-A cells proliferation and migration in vitro

**Figure 7:**
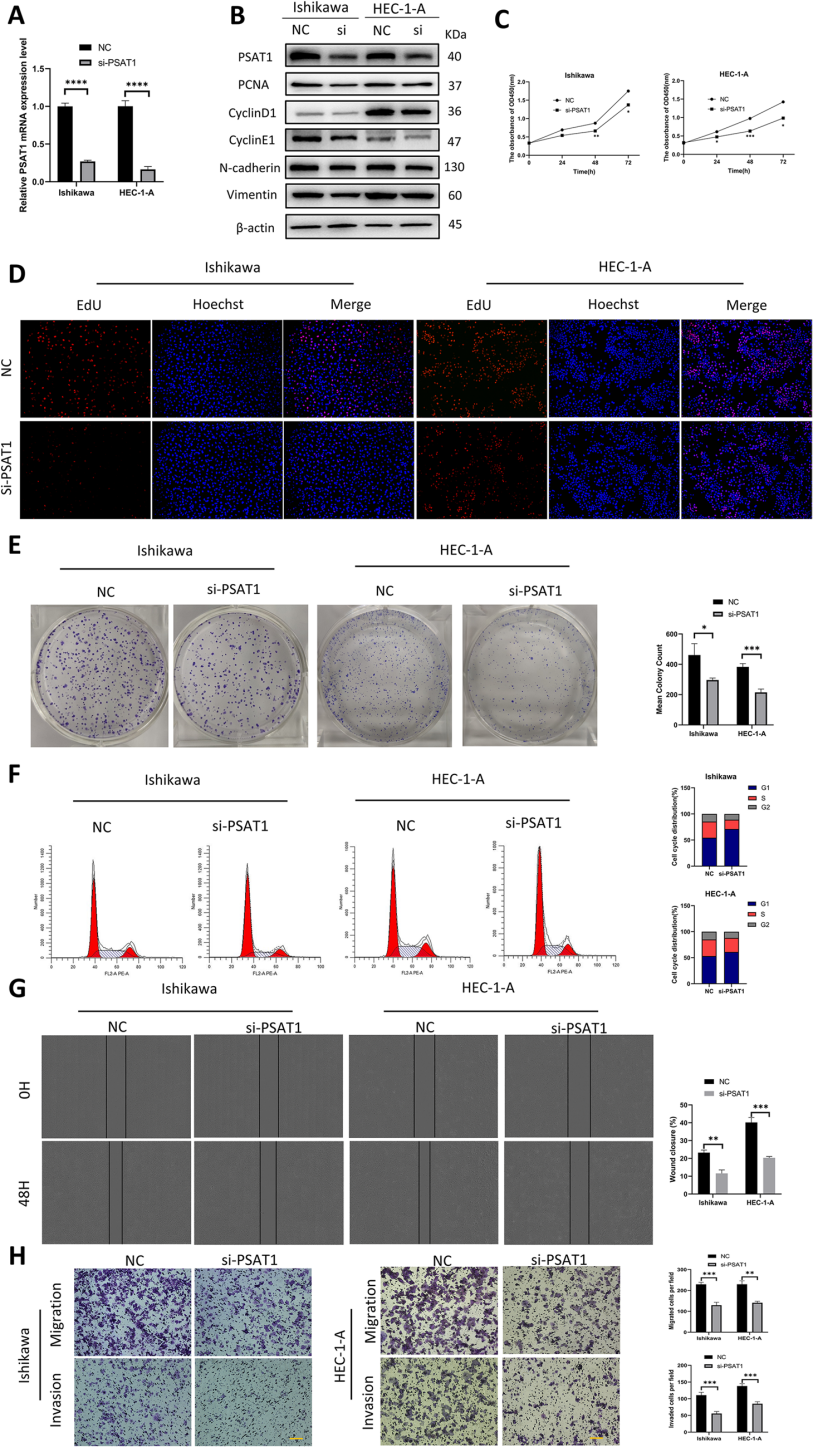
PSAT1 promotes Ishikawa and HEC-1-A cells proliferation and migration. (**A**) qPCR and (**B**) western blotting showed the PSAT1 knockdown efficiency and the protein expression level of PCNA, CyclinD1, CyclinE1, N-cadherin and Vimentin in Ishikawa and HEC-1-A cells. Three independent experiments were performed; representative results were shown. The blots were cut prior to hybridization with antibodies according to the molecular size of the target gene. PSAT1 protein was detected around 40 kDa. PCNA protein was detected below 40 kDa and above 35KDa. CyclinD1 protein was detected below 40 kDa and above 25KDa. CyclinE1 protein was detected below 55 kDa and above 40KDa. N-cadherin protein was detected below 150 kDa and above 100KDa. Vimentin protein was detected below 70 kDa and above 55KDa. Data normalized using β-actin. β-actin protein was detected below 55 kDa and above 40 KDa. Original blots were presented in Supplementary Fig. 1. (**C**) CCK8, (**D**) EdU and (**E**) clone formation assays demonstrated that PSAT1 promotes Ishikawa and HEC-1-A cells proliferation. (**F**) Cell cycle analysis by flow cytometry. (**G**) Wound healing assay of Ishikawa and HEC-1-A cells. (**H**) Transwell assay to detect the migration and invasion of Ishikawa and HEC-1-A cells. Scale bar: 100 μm. *p < 0.05, **p < 0.01, ***p < 0.001, ****p < 0.0001.

To further validate the role of PSAT1 in UCEC, we performed several functional experiments. qPCR and western blotting confirmed that PSAT1 was successfully knocked down in Ishikawa and HEC-1-A cells. After knocking down PSAT1, the protein expression level of proliferating nuclear antigen (PCNA) decreased (Fig. 7A,B). The CCK8, EdU and clone formation assays confirmed that PSAT1 knockdown inhibited proliferation in Ishikawa and HEC-1-A cells (Fig. 7C–E). According to flow cytometry, the number of Ishikawa and HEC-1-A cells in the S and G2 phases decreased, and more cells aggregated in the G1 phase after PSAT1 knockdown (Fig. 7F). In addition, the results of western blotting in Fig. 7B showed that the protein expression levels of CyclinD1 and CyclinE1 decreased after PSAT1 was knocked down. CyclinD1 and CyclinE1 are two key proteins that regulate G1 phase and the transition from G1 phase to S phase of cells^31^. These results suggested that PSAT1 might mediate the G1 phase arrest of cells in UCEC. These experiments demonstrated that PSAT1 plays a pro-proliferative role in UCEC. PSAT1 knockdown also reduced cell migration and invasion in Wound healing and Transwell assays (Fig. 7G,H). Epithelial-mesenchymal transition (EMT) plays an important role in the migration and invasion of epithelial tumor cells^32^. We detected the protein expression levels of EMT-related makers (N-cadherin and Vimentin) in Ishikawa and HEC-1-A cells by western blotting. The results showed that the protein expression levels of N-cadherin and Vimentin were reduced after PSAT1 was knocked down (Fig. 7B). These results suggested that PSAT1 might promote cell migration and invasion through EMT in UCEC. Overall, PSAT1 can influence the progression of UCEC by regulating cell proliferation, migration, and invasion.

## Discussion

UCEC is one of the major causes of poor health in women. The prognosis of early-stage UCEC is often optimistic, but that of most advanced is poor and can even result in death^33^. Therefore, it is important to identify early diagnostic markers for UCEC^34,35^. There has evidence that PSAT1 acts as an oncogene in many tumors^36–38^ and that it plays an important role in the development of various cancers^39^. In our study, PSAT1 was significantly overexpressed in UCEC from TCGA. Furthermore, UCEC patients with higher expression of PSAT1 had a worse prognosis than those with lower expression of PSAT1, and the upregulated expression of PSAT1 was related to the clinical stage, age, histological type, and weight of UCEC. In the ROC curve analysis of PSAT1, the AUC value for the diagnosis of UCEC was high. PSAT1 may therefore prove useful as a potential biomarker for the diagnosis of UCEC.

To further explore the reasons for the differences in UCEC prognosis, the DEGs was analyzed between patients with high and low PSAT1 expression. The results revealed that in UCEC patients with high PSAT1 expression, 32 genes were up-regulated, and 116 genes were down-regulated. Furthermore, GO enrichment analysis was performed on the 148 DEGs, and the functions were mainly concentrated in metabolic processes, immune system processes and growth. UCEC patients are often obese and have metabolic abnormalities^40,41^. The results of GO enrichment analysis were consistent with this. These results showed that PSAT1 may participate in the metabolic regulation of UCEC. In addition, KEGG enrichment analysis was used to identify the crucial pathways associated with PSAT1 in UCEC revealing a significant enrichment of cell cycle and cellular senescence. From these results, we speculated that PSAT1 might affect the prognosis of UCEC by affecting the cell cycle, cell growth, and immune system processes; indeed, several of our in vitro functional experiments demonstrated that PSAT1 affects UCEC progression by regulating cell proliferation, migration and invasion.

Tumor progression is often closely associated with the surrounding immune microenvironment^42–47^, and abnormal immune infiltration in patients with UCEC has been widely reported^1,48–50^. GO enrichment analysis revealed that PSAT1 might be involved in immune infiltration; therefore, we conducted ssGSEA to explore the relationship between PSAT1 and the immune cells. Th2 cells promote macrophage differentiation into M2 macrophages, which are a pro-tumor subtype^48^. Astragaloside-IV can slow down the progression of lung cancer by partially blocking the M2 polarization of macrophages through the AMPK signaling pathway^51^. In our study, there was a positive correlation between PSAT1 mRNA expression levels and Th2 cell infiltration. This may explain the poor prognosis of patients with UCEC with high expression levels of PSAT1. In addition, our research found a negative correlation between PSAT1 mRNA expression levels and the infiltration of Th17 cells, which can recruit and activate neutrophils and are associated with improved survival^52,53^. According to these results, PSAT1 might affect the prognosis of patients with UCEC by affecting immune infiltration of Th2 and Th17 cells. Therefore, PSAT1 may be a potential target for immunotherapy in UCEC.

We predicted the possible upstream miRNAs of PSAT1 in UCEC using the StarBase database to further explore the regulatory mechanism of PSAT1. The results revealed that PSAT1 expression was significantly negatively correlated with two miRNAs (miR-195-5P and miR-497-5p) and PSAT1 expression, where the expression patterns of these two miRNAs in UCEC were opposite to that of PSAT1. This was consistent with the mechanism whereby miRNAs regulate target genes^54–56^. Furthermore, survival analysis showed that the expression levels of hsa-miR-195-5P and hsa-miR-497-5p were significantly correlated with the prognosis of UCEC. In order to clarify which miRNAs regulate the function of PSAT1, we transfected a miR mimic/inhibitor into Ishikawa and HEC-1-A cells to achieve the effect of miRNA overexpression or knockdown. The results showed that PSAT1 expression was negatively regulated by miR-195-5P but not by miR-497-5P. These results suggest that miR-195-5P is an upstream regulator of PSAT1.

Several studies revealed the crucial role of transcription factors in cancers^57,58^. We used AnimalTFDB 4.0 (http://bioinfo.life.hust.edu.cn/AnimalTFDB4/#/) to predict the transcription factors that bind to PSAT1. The spearman results of transcription factors with p < 0.05 and cor > 0.3 are shown in the Supplementary Table 1. These transcription factors with significant correlation with PSAT1 may have significant implications for the mechanism by which PSAT1 regulates UCEC progression. We will conduct a more in-depth study of these transcription factors in the future.

This study had several limitations. First, abnormal immune cell infiltration in UCEC requires further verification. Furthermore, the mechanism by which the miR-195-5P/PSAT1 axis regulates UCEC prognosis requires further investigation. We aim to conduct relevant research in the future to address these limitations.

## Conclusions

Our study showed that PSAT1 was significantly overexpressed in UCEC and was closely related to its prognosis. Moreover, PSAT1 could be used as an independent predictor for UCEC diagnosis and affect UCEC prognosis by regulating cell proliferation and migration. Furthermore, we found that miR-195-5P is the upstream regulatory gene of PSAT1. The prognosis of UCEC is also influenced by immune infiltration by Th2 and Th17 cells. Thus, PSAT1 may be an effective target for UCEC diagnosis and immunotherapy in the future.

## Methods

### Study design and samples acquisition

We downloaded RNA sequence data from TCGA (https://portal.gdc.cancer.gov/). Level 3 HTSeq-FPKM data were converted to TPM for subsequent analysis, and a log_2_ conversion was performed. UCEC patients were classified into two groups, high- and low-PSAT1 expression patients according to their median expression levels of PSAT1. Unpaired samples were analyzed using Wilcoxon rank-sum test, while paired samples were analyzed using paired sample t-test. The Kaplan–Meier plotter was used to construct the survival curves. The CPTAC and the HPA databases were used to explore the expression of PSAT1 in UCEC versus normal endometrial tissue at the protein level.

### Analysis of DEGs between the high- and low-PSAT1 expression groups

DEGs between high- and low-PSAT1 expression patients from TCGA datasets were identified using the limma package in the R software. The adjusted P-value < 0.05 and |Fold Change|> 1.5 were considered as the threshold for the differential expression of mRNAs.

### GO enrichment analysis

The Metascape database2.0 and online tool (http://metascape.org) were used for functional enrichment analysis. In this study, significance was defined as an enrichment factor > 1.5, a minimum count of 3, and a P-value < 0.01.

### KEGG enrichment analysis

The clusterProfiler, enrichplot, and ggplot2 R packages were used for KEGG enrichment analysis. A P-value < 0.05 was regarded as significantly enriched.

### Immune infiltration analysis by ssGSEA

To elucidate the correlation between PSAT1 and the level of immune cell infiltration, ssGSEA from the GSVA package [version 1.34.0] was used^59^. The markers of the 24 types of immune cells originated from a previous immune article^60^. The correlation between the immune cell infiltration level and PSAT1 was determined using Spearman’s rank correlation.

### Candidate miRNA prediction

Interactions between miRNAs and PSAT1 were predicted using StarBase (http://starbase.sysu.edu.cn/). StarBase was also used to conduct a correlation analysis of miRNA-PSAT1 in UCEC. The expression levels of candidate miRNAs in UCEC were also analyzed. Statistical significance was set at p < 0.05.

### Cell culture

The two human endometrial cancer cell lines, Ishikawa and HEC-1-A were purchased from Shanghai Jinyuan Biotechnology Co., Ltd. and Procell (Wuhan, China), respectively. DMEM basic (GIBCO, USA) medium containing 10% fetal bovine serum and 1% penicillin and streptomycin was used for the culture of Ishikawa cells, and McCoy's 5A medium containing 10% fetal bovine serum and 1% penicillin and streptomycin was used for the culture of HEC-1-A cells in a 37 °C cell incubator with 5% carbon dioxide.

### MiR mimic/inhibitor and siRNA transfection

We purchased the miR-195-5P mimic/inhibitor and miR-497-5P mimic/inhibitor from RiboBio (Guangzhou, China), and Tsingke Biotechnology Co., Ltd designed and synthesized the siRNAs. The miR mimic/inhibitor and siRNA sequences are listed in Supplementary Table 2. Lipofectamine 2000 Reagent (Invitrogen, USA) was used for miR mimic (50 nM), miR inhibitor (100 nM), and siRNA (50 nM) transfection in Ishikawa and HEC-1-A cells. At 48–96 h after transfection, transfection efficiency was measured. All operations were performed according to the manufacturer’s instructions.

### Real-time quantitative PCR

Total RNA was extracted using an RNA-Quick Purification Kit (ESscience, China). The PrimeScript™ RT Reagent Kit with a gDNA Eraser (Takara, Japan) and SYBR Green™ Premix Ex Taq™ II (Takara, Japan) were used for reverse transcription and real-time PCR. Primer sequences are shown in Supplementary Table 2.

### Western blot

RIPA lysis buffer (Beyotime, China) containing a protease and phosphatase inhibitor mixture was used to extract proteins from Ishikawa and HEC-1-A cells. A BCA Protein Assay Kit (Solarbio, China) was used to detect protein concentrations. We separated proteins with sodium dodecyl sulfate–polyacrylamide gel (SDS-PAGE) and then transferred them to PVDF membranes. After blocking with 5% non-fat milk for 1.5 h at room temperature, the membranes were incubated with the primary antibody overnight at 4 °C. We used the following primary antibodies, anti-β-actin (Servicebio, China), anti-PCNA (Proteintech, China), anti-PSAT1 (Proteintech, China), anti-N-cadherin (Proteintech, China), anti-Vimentin (Proteintech, China), anti-CyclinD1 (ZenBio, China) and anti-CyclinE1 (ZenBio, China). After washing the membranes with TBST three times, membranes were incubated with a secondary antibody (ZSGB Bio, China) for 1.5 h at room temperature, and then a chemiluminescence imaging system (Clinx S6, China) was used to visualize the protein bands.

### Cell proliferation assays

About 3000 cells per well were seeded in a 96-well plate, and cell proliferation was evaluated using the CCK-8 (APExBIO, USA) assay according to the manufacturer’s protocol. Two hours after incubation with CCK-8, the absorbance was measured at OD450 using a Multifunctional Microplate Reader (Thermo Fisher Scientific, USA).

### EdU assay

A Cell-Light EdU Apollo In Vitro Kit (RiboBio, China) was used according to the manufacturer’s protocol. The cells were then observed and photographed using a fluorescence microscope (Nikon ECLIPSE Ti, Japan).

### Clone formation assays

About 1000 cells per well were seeded in a 6-well plate, and after 10–14 d of culture, the cells were fixed with 4% paraformaldehyde (Servicebio, China) and stained with crystal violet (Solarbio, China). Finally, the clones were photographed and counted.

### Flow cytometry

Forty-eight hours after the cells were transfected in a 6-well plate, they were collected from each well. After washing the cells twice with PBS, 75% alcohol was added slowly to fix the cells. The tube was shaken while alcohol was added to prevent cell aggregation. Finally, the cells were stained with PI (BD Biosciences, USA) for 10 min and subjected to flow cytometry for cell cycle detection.

### Wound healing assay

A 6-well plate was used to culture cells and the cells were scratched using a 10 μL pipette tip when the cell density reached approximately 90%. The cell culture medium was then replaced with a serum-free basal medium. The scratches were photographed at 0 and 48 h, and Image J software was used for image quantification.

### Transwell assay

Transwell assays were performed using 8-µm Transwell chambers (Biofil, China) in 24-well plates. In migration assay, lower chambers were filled with 500 μL medium containing 10% serum, while upper chambers were seeded with approximately 40,000 cells resuspended in 200 μL of serum-free basal medium. Regarding invasion assay, before adding cells to the transwell chambers, they were evenly covered with Matrigel (Corning, USA) (50 μL per chamber). Then the plates were placed in a 37 ℃ incubator and incubated for 2 h. Subsequent steps are the same for migration assay and invasion assay. After incubating for 24 h, cells that migrated to the outside of the chamber were fixed with 4% paraformaldehyde for 20 min and stained with crystal violet (Beyotime, China) for 30 min. The cells were then photographed and counted.

### Statistical analysis

We used the paired sample t-test and Wilcoxon rank-sum test to determine the expression levels of PSAT1 in paired and non-paired samples. The Kaplan–Meier plotter was used to construct the survival curves. We used the ROC curve to explore the diagnostic value of the expression level of PSAT1 based on the pROC package. The p values of all results were bilateral, with a significance level of 0.05. Data analysis was performed using R version 3.6.3 and GraphPad Prism 8.0.2. All data in this study are presented as means ± SD, and at least three independent replicates were performed for all experiments.

## Supplementary Information

Supplementary Information.

## Data Availability

All data are available upon request from the corresponding author.
