# Supplementary material for: Downregulation of PSAT1 inhibits cell proliferation and migration in uterine corpus endometrial carcinoma
Source: Sci Rep. 2023 Mar 11;13:4081. doi: 10.1038/s41598-023-31325-0 (PMC10008565; doi:10.1038/s41598-023-31325-0)

Downregulation of PSAT1 inhibits cell proliferation and migration in uterine corpus endometrial carcinoma

Min Wang[1], **Song Yue**[1], **Zhu Yang**[1*]

[1]Department of Gynecology and Obstetrics, the Second Affiliated Hospital of Chongqing Medical University, Chongqing, 400010, China
[*]Corresponding. yangzhu@hospital.cqmu.edu.cn

Supplementary Table. 1. Transcription factor correlation analysis of PSAT1.

TF	immuneGene	cor	pvalue	Regulation
PSAT1	STAT1	0.376077189	0	postive
PSAT1	ZNF675	0.352401445	6.04E-19	postive
PSAT1	REST	0.337295611	6.47E-17	postive
PSAT1	GMEB2	0.327941889	5.72E-16	postive
PSAT1	ZFP64	0.302269766	1.16E-13	postive
PSAT1	ZNF770	0.301272121	1.40E-13	postive
PSAT1	HEY2	-0.309280652	2.90E-14	negative
PSAT1	FOXP1	-0.345798786	6.75E-18	negative
PSAT1	SPDEF	-0.350894622	1.20E-18	negative
PSAT1	RARA	-0.430761856	0	negative

Supplementary Table. 2. Sequences used in this study.

Genes	Sequences
miR-195-5p	RT:
	GTCGTATCCAGTGCAGGGTCCGAGGTATTCGCACTGGATACGACGCCAAT
	F: CCTCGAGCTAGCAGCACAGAAA
	R: ATCCAGTGCAGGGTCCGAGG
miR-497-5p	RT:
	GTCGTATCCAGTGCAGGGTCCGAGGTATTCGCACTGGATACGACACAAAC
	F: AACAGTGCAGCAGCACACTGT
	R: ATCCAGTGCAGGGTCCGAGG
Homo-U6	F: CTCGCTTCGGCAGCACA
	R: AACGCTTCACGAATTTGCGT
miR-195-5P mimic	F: UAGCAGCACAGAAAUAUUGGC
	R: AUCGUCGUGUCUUUAUAACCG
miR-195-5P inhibitor	AUCGUCGUGUCUUUAUAACCG
miR-497-5P mimic	F: CAGCAGCACACUGUGGUUUGU
	R: GUCGUCGUGUGACACCAAACA
miR-497-5P inhibitor	GUCGUCGUGUGACACCAAACA
NC mimic	F: UUUGUACUACACAAAAGUACUG
	R: CAGUACUUUUGUGUAGUACAAA
NC inhibitor	CAGUACUUUUGUGUAGUACAAA
PSAT1	F: CTAAGCGTTGGTCTGGCAGGAAG
	R: AGAAGTGGAGAGCAGATGGAGGAG
si PSAT1	F: GCUGUUCCAGACAACUAUA
	R: UAUAGUUGUCUGGAACAGC
GAPDH	F: CGACCACTTTGTCAAGCTCA
	R: CCCTGTTGCTGTAGCCAAAT

Supplementary Fig. 1. Original blots

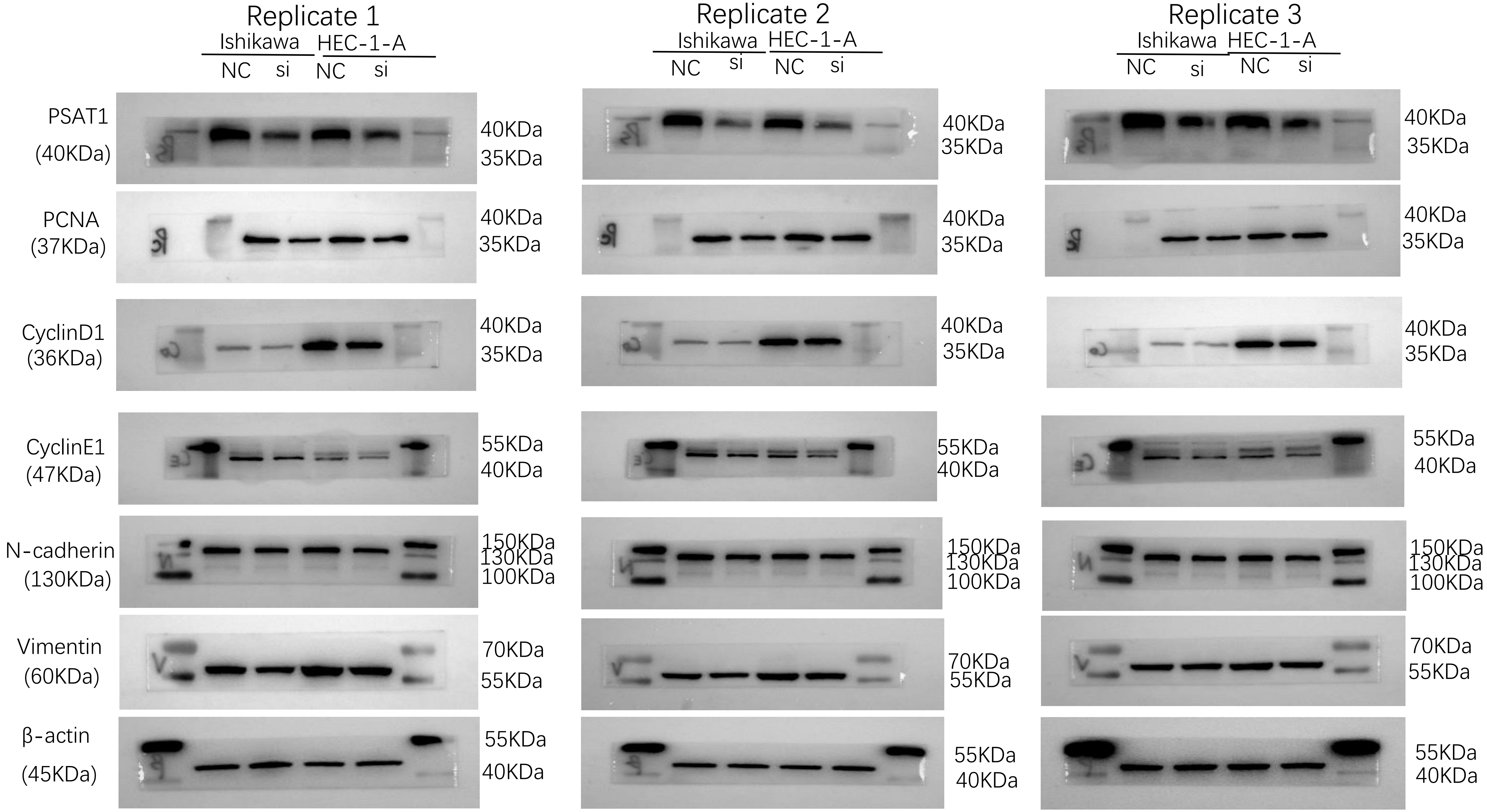

Supplement: Supplementary file 1 — Supplementary Information. [file 41598_2023_31325_MOESM1_ESM.pdf]
